# Phenotypic Diversity in Leaf Cuticular Waxes in *Brassica carinata* Accessions

**DOI:** 10.3390/plants12213716

**Published:** 2023-10-29

**Authors:** Pernell Tomasi, Hussein Abdel-Haleem

**Affiliations:** USDA-ARS, US Arid-Land Agricultural Research Center, 21881 North Cardon Lane, Maricopa, AZ 85138, USA

**Keywords:** *Brassica carinata*, cuticle, drought, cuticular wax, wax classes, wax components, stress tolerance

## Abstract

*Brassica carinata* has received considerable attention as a renewable biofuel crop for semi-arid zones due to its high oil content and polyunsaturated fatty acids contents. It is important to develop new drought-resistant cultivars of *B. carinata* production to expand its areas into more arid regions. The accumulation of leaf cuticular wax on plant surfaces is one mechanism that reduces non-stomatal water loss, thus increasing drought resistance in plants. To explore phenotypic variations in cuticular wax in *B. carinata*, leaf waxes were extracted and quantified from a diversity panel consisting of 315 accessions. The results indicate that the accessions have a wide range of total leaf wax content (289–1356 µg dm^−2^), wax classes, and their components. The C_29_ and C_31_ homologues of alkanes, C_29_ ketone homologue, C_29_ secondary alcohol, and C_30_ aldehyde were the most abundant leaf waxes extracted from *B. carinata* accessions. The high heritability values of these waxes point to the positive selection for high wax content during early generations of future *B. carinata* breeding programs. Positive correlation coefficients, combined with the effects of these waxes on leaf wax content accumulation, suggest that modifying specific wax content could increase the total wax content and enhance cuticle composition. The identified leaf wax content and compositions in *B. carinata* will lead to the future discovery of wax biosynthetic pathways, the dissection of its genetic regulatory networks, the identification of candidate genes controlling production of these waxes, and thus, develop and release new *B. carinata* drought-tolerant cultivars.

## 1. Introduction

Vegetable oils from several traditional crops, including soybean (*Glycine max* (L.) Merr), corn (*Zea mays* L.), canola (*Brassica napus* subsp. *napus*), and sunflower (*Helianthus annuus* L.), as well new industrial crops such as carinata *(Brassica carinata* A. Braun), camelina (*Camelina sativa* L.), pennycress (*Thlaspi arvense* L.), crambe (Crambe abyssinica R.E.Fr.), jojoba (*Simmondsia chinensis* (Link) C. K.) and jatropha (*Jatropha curcas* L.), are used to meet the increasing demand for biofuels, such as a biodiesel source. Drenth et al. [1] concluded that oils extracted from industrial crops have very similar engine performance to traditional oils crops. An ideal biofuel crop should have low agricultural inputs, a high oil content, a high polyunsaturated fatty acid content, be compatible with existing farm equipment and infrastructure, be tolerant to low-input growth conditions, have low irrigation requirements, have definable growth seasons, and maintain uniform seed maturation rates [2]. There is a pressing need to develop high-yielding, non-food oil crops that can be cultivated in underutilized farming areas, thus avoiding the food vs. fuel debate. Based on these criteria, plants such as carinata, camelina, and pennycress promise non-food crops for low-input and marginal agricultural systems in the U.S.

*Brassica carinata* A. Braun is a species native to Ethiopian highlands and has recently become the subject of increasing interest due to its high oil and erucic fatty acid content (~40%). *Brassica carinata* oil can be refined into biofuels that meet the specifications of petroleum-based fuels [3,4,5]. This crop has better agronomic performance (seed yield, resistance to various biotic stresses and diseases, tolerant to abiotic stresses, and resistance to pod shattering) compared to other brassicas, especially camelina and canola, in semi-arid areas of the Northern Great Plains, Canadian Prairies, and Southern Europe [6,7,8,9,10,11,12]. *Brassica carinata* could prove more adaptable to marginal environmental conditions that require crops with low irrigation requirements to grow [13]. These growing conditions make *B. carinata* an ideal crop for marginal lands in the wheat-producing regions of the Northern Great Plains (NGP) of the U.S. Historically, the semi-arid regions of the NGP were dominated by monoculture cereal cropping systems that include a 14-month fallow period and extensive use of mechanical tillage [14].

Abiotic stresses are estimated to reduce crop yields to less than half of the gain under ideal growing conditions [15]. Notably, vascular plants have evolved mechanisms to survive abiotic stress. However, the underlying molecular mechanisms are not well understood in most agricultural crops. Plants adapted to avoid tissue dehydration possess more efficient root systems underground that increase soil water extraction, and/or exhibit highly efficient above-ground systems to reduce water evaporation, such as control over stomatal conductance, modification of solar radiation absorption, and/or modification of cuticle water permeability [16]. Non-stomatal water loss is primarily controlled by the cuticle, an extracellular lipophilic polymer that covers and protects the aerial organs of plants. The cuticle also provides protection from environmental stresses, such as pathogens, insects [17,18], supra-optimal air temperature, and high solar radiation [18,19,20,21,22,23]. The cuticle consists of two lipid classes; the non-polymerized cuticular wax components and cutin polyester layers. Cuticular waxes are mostly saturated very-long-chain (C_20_–C_34_) fatty acids that occur as epicuticular and intra-cuticular lipids. Cutin monomers consist of C_16_ and C_18_ fatty acid derivatives (e.g., hydroxy fatty acids and dicarboxylic acids) linked primarily by ester bonds to form a polyester matrix. Two major pathways involved in the synthesis of cuticular wax includes the primary alcohol-forming pathway, which produces very long-chain alcohols and wax esters, and the alkane-forming pathway, which produces aldehydes, alkanes, secondary alcohols, and ketones [18]. Additional components of waxes include cyclic triterpenes and other aliphatic components, some of which can account for a significant proportion of total wax composition [24]. Among the 40 leaf wax components identified in Camelina species and the *Camelina sativa* diversity panel [25,26], primary alcohols (mainly C_24_, C_26_, and C_28_) and alkanes (mainly C31) are the predominant leaf wax classes, followed by wax esters, fatty acids, and aldehydes. These wax components show wide phenotypic variations among Camelina accessions. Previous studies have shown that there is a positive correlation between drought resistance and wax accumulation deposited on the leaf surface in plants, including Arabidopsis [20], tobacco [27], peanut [28], soybean [29], wheat [30,31], and cotton [32]. Wax composition often varies significantly among plant species and organs [33,34,35], developmental stages [33,36], and in response to environmental conditions [21,37]. As leaf wax components vary among species, organs, and in response to environmental stresses, it is expected that the genes involved in wax accumulation also vary and belong to different pathways. For example, under drought stress conditions, a transgenic camelina line overexpressing a transcription factor MYB96 accumulated leaf wax by 52% more than the null control line [38].

The nature and inheritance of *B. carinata*’s response to abiotic stresses, such as drought, is unknown. The goal of this current research is to explore the variation in leaf wax composition in *B. carinata*. The objectives of the current study are to characterize the phenotypic variations in *B. carinata* leaf wax content and compositions and estimate the heritability and genetic components of the accumulations of these waxes.

## 2. Results

The first step in expanding our understanding of leaf wax-accumulating mechanisms and identifying candidate genes controlling wax biosynthetic pathways in *B. carinata* is to identify and quantify leaf wax content, waxes classes and components in large diverse populations. The analysis of 315 *B. carinata* accessions, collected from its area of origin and different growing areas, revealed wide variations in wax content, classes, and components (Supplementary Table S1).

**Figure 1:**
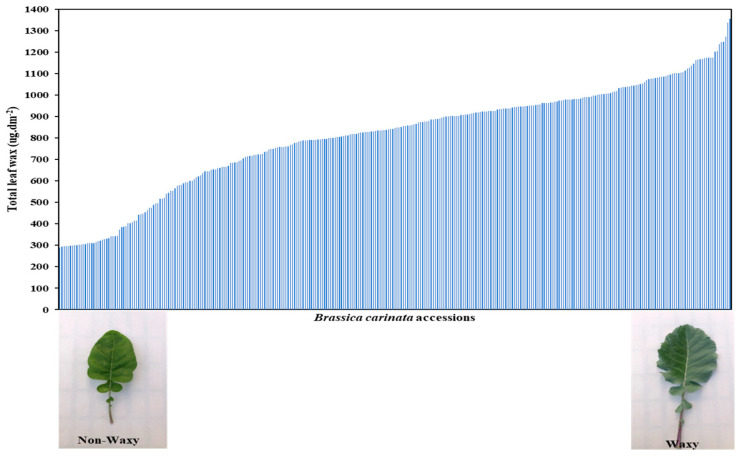
Total leaf wax BLUP values of 315 *Brassica carinata* accessions.

The *B. carinata* diversity panel showed a wide range of total wax contents, where the highest accession, collected from the Bonga region in Ethiopia, accumulated 1356 µg dm^−2^ of total wax on the leaf surface, while one of the lowest accessions, collected from Sweden, accumulated 289 µg dm^−2^ (Table 1, Figure 1). The extracted leaf waxes from *B. carinata* consist of seven main classes.

Among these classes, alkanes and ketones accounted for 77% of the accumulated leaf wax; primary alcohols and secondary alcohols accounted for 15%, and aldehydes, methyl alcohols, free fatty acids, and wax esters accounted for around 8% (Table 1). *B. carinata’s* alkane content ranged from 145 µg dm^−2^ to 637 µg dm^−2^. The C_29_ and C_31_ alkanes accounted for 35% and 12% of the total leaf waxes in *B. carinata*, respectively (Table 1). The C_29_ ketone accounted for 27% of the total wax accumulated on *B. carinata* leaves, making it the second most abundant wax after C_29_ alkane (Table 1). *B. carinata* accessions showed a wide phenotypic variation, with a 79 µg dm^−2^ to 453 µg dm^−2^ range for C_29_ Ket (Table 1). Primary alcohols contributed 8% to the total waxes (Table 1), with *B. carinata* accessions ranging from 48 µg dm^−2^ to 86 µg dm^−2^ in primary alcohols. Among primary alcohols in *B. carinata*, C_26_ Alc and C_28_ Alc were the predominant molecular species, together accounting for 7% of total waxes (Table 1). Secondary alcohols accounted for 7% of the total leaf wax in *B. carinata*, with the lowest amount at 16 µg dm^−2^ and the highest amount at 87 µg dm^−2^ (Table 1). The prominent secondary alcohol is C_29_ Alc-2, accounting for 5% of the total leaf waxes (ranging from 15 µg dm^−2^ to 75 µg dm^−2^). The C_30_ Ald (an aldehyde wax component) accounted for 7% of the total wax, with a range from 20 µg dm^−2^ to 52 µg dm^−2^. Other wax classes each accounted for less than 5% of the total wax content but still showed significant phenotypic variation within each class; where methyl alcohols ranged from 13 µg dm^−2^ to 41 µg dm^−2^, free fatty acids ranged from 8 µg dm^−2^ to 17 µg dm^−2^, and C_43_ WE (a wax ester component) ranged from 7.28 µg dm^−2^ to 7.94 µg dm^−2^ (Table 1). Even though these wax components have a small contribution to the total wax content, they could play a role in stress tolerance.

**Figure 2:**
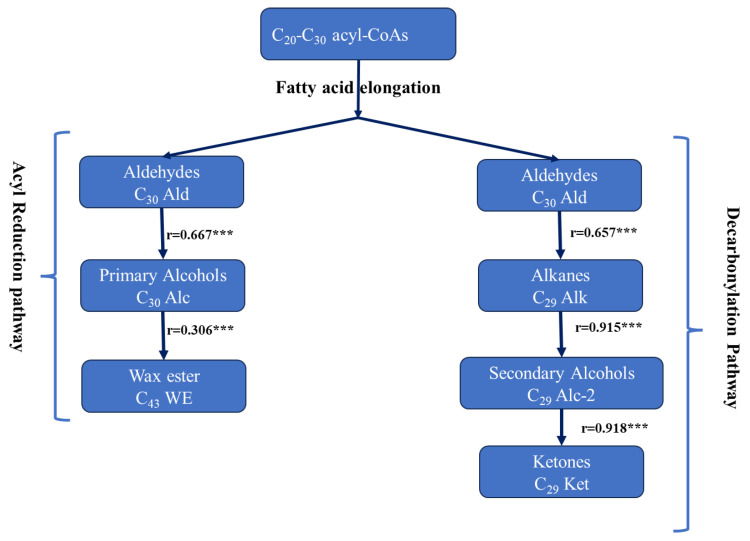
Correlation coefficients for selected wax components in cuticular wax biosynthesis. *** is the significance levels at *p* < 0.001.

Cuticular wax classes are produced via two main pathways: acyl reduction and decarbonylation [19,34,39,40]. In the decarbonylation pathway (alkane-forming pathway), aldehydes are produced from VLCFA precursors, followed by aldehyde decarbonylation to produce alkanes that may be converted into ketones via secondary alcohols. Highly significant positive correlation coefficients were observed between C_30_ Ald and C_29_ Alk (r = 0.658), C_29_ Alk and C_29_ Alc-2 (r = 0.915), and C_29_ Alc-2 and C_29_ Ket (r = 0.918) (Figure 2, Supplementary Table S2). These findings suggest that the decarbonylation pathway is the main pathway for wax biosynthesis in *B. carinata.* In the acyl reduction pathway (alcohol-forming pathway), primary alcohols are produced by reducing very long-chain fatty acid (VLCFA) precursors to produce aldehydes, primary alcohols, and wax esters via the esterification of fatty acids and primary alcohols. In *B. oleracea*, it was shown that acyl-CoA reductase converts fatty-CoA into aldehydes, which are then reduced by aldehyde reductase to produce primary alcohols [41].

Significant positive correlation coefficients were observed between C_30_ Ald and C_29_ Alc (r = 0.812), C_29_ Alc and C_43_ WE (r = 0.439), and C_30_ Ald and C_43_ WE (r = 0.474) (Figure 2, Supplementary Table S2). These observations suggest that primary fatty alcohols, wax esters, and free fatty acids have a lesser effect on the variations in *B. carinata* leaf waxes. Tassone et al. [42] obtained similar results in *B. napus* and suggested that both pathways are coupled in their effects to produce and accumulate leaf waxes.

**Figure 3:**
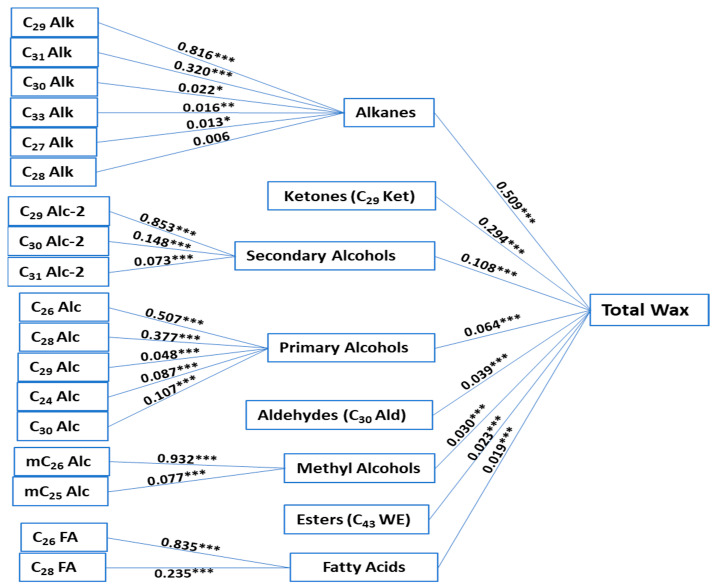
Direct effects of the total wax content related to wax classes and components. *, **, *** are the significance levels at *p* < 0.05, *p* < 0.01, and *p* < 0.001, respectively.

Highly significant correlation coefficients were detected between total wax and free fatty acids (r = 0.602), primary alcohols (r = 0.619), secondary alcohols (r = 0.947), methyl alcohols (r = 0.838), alkanes (r = 0.983), wax esters (r = 0.0.465), aldehydes (r = 0.653), and ketones (r = 0.970) (Table S2). These correlations could indicate that these leaf wax compounds are dependently regulated. To gain a deeper understanding of the correlations among wax classes in *B. carinata*, path analysis was conducted to partition the correlation coefficients for total wax accumulation and its wax classes into direct and indirect effects contributing to total wax accumulation. Alkanes had the highest positive direct effect on total wax accumulation, followed by C_29_ Ket and secondary alcohols with 0.509, 0.294, and 0.108, respectively (Figure 3, Supplementary Table S3). Among the detected alkanes, C_29_ Alk and C_31_ Alk had indirect positive effects on total wax accumulation, displaying 0.415 and 0.163, respectively (with direct positive effects of 0.816 and 0.320 on alkanes accumulation). C_29_ Alc-2 and C_29_ Alc-2 had the highest positive indirect effects, with 0.092 and 0.016, respectively (with direct effects of 0.853 and 0.148 on secondary alcohols accumulation). The detected C_30_ Ald had a positive direct effect of 0.039 on total wax accumulation. Even though total free fatty acid was highly correlated with total wax (r = 0.602), its direct effect on accumulation was the lowest among wax classes (0.09). The path analysis results indicated that the alkane-forming pathway exhibits a higher influence on leaf wax accumulation in *B. carinata*, with C_29_ Alk, C_29_ Ket, C_31_ Alk, C_29_ Alc-2, and C_30_ Ald being the most prominent waxes (from the higher effect to the lower effect).

**Figure 4:**
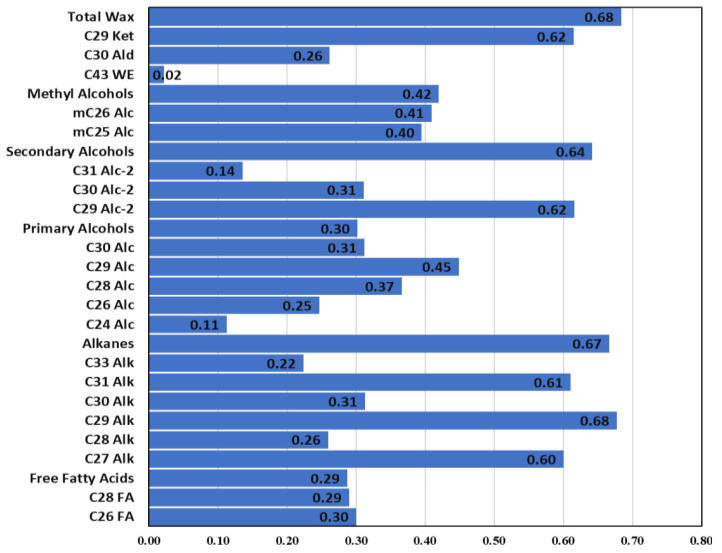
Broad-sense heritability values (H^2^) of total wax content, wax classes, and components.

High heritability estimates indicate the feasibility of selecting a trait of interest during the early generations of breeding programs [34]. The total wax content in the current study showed a high heritability estimate (H^2^ = 0.68, based on broad-sense heritability), indicating a high genetic component controlling leaf wax content in *B. carinata*. For wax classes and components, heritability values ranged from 0.02 (C_43_ Wax ester) to 0.68 (C_29_ Alkane) (Figure 4), demonstrating the variable environmental effects on the inheritance of these waxes. In general, alkanes, secondary alcohols, and C_29_ ketone showed high heritability values, each greater than H^2^ = 0.65, while secondary and methyl alcohols showed moderate heritability values (H^2^ is more than 30 and less than 0.60).

## 3. Discussion

Plant adaptation to drought stress mechanisms can be grouped into four main categories: escape, avoidance, tolerance, and recovery [43]. The avoidance mechanism generally increases a plant’s ability to delay tissue dehydration when soil moisture depletes. Accumulating cuticular wax on plant surfaces is one of the strategies to reduce nonstomatal water loss under abiotic stresses [43,44]. A large diversity panel of *B. carinata* accessions collected from different geographical areas was used to explore the phenotypic diversity in leaf waxes, aiming to identify candidate genes controlling wax biosynthesis pathways. To date, there have been few reports describing leaf wax structure on the *B. carinata* leaf surface [45,46,47]. This is the first report on identifying wax classes and components in *B. carinata*. The *B. carinata* diversity panel showed a wide range of total wax contents, in similar fashion to *B. napus* [42], with seven main wax classes extracted and classified. In *B. carinata*, alkanes constituted the most abundant wax class. Alkanes positively increased under drought stresses on the leaves of alfalfa [48], Arabidopsis [20,49], sesame [50] soybean [29], and *Populus euphratica* [51]. The C_29_ Alk accounted for up to 45% of the waxes accumulated on *B. napus* leaves [42]. Li et al. [52] found that the C_29_ Alk and C_31_ Alk contents increased in watermelon leaves in response to drought stress. The abundant C_29_ Alk accounted for 35% of the total leaf waxes in *B. carinata,* indicating the possible role of C_29_ Alk in abiotic stress tolerance. The second most abundant wax class was ketones, mainly represented as C_29_ Ket wax. An analysis of 504 *B. napus* accessions indicated that the C_29_ Ket ranged from 200 µg dm^−2^ to 574 µg dm^−2^ [42]. Ketones were found to be a major wax class in species such as B. oleracea and Allium porrum, with 31% and 52% of the total wax [53]. Kosma et al. [20] found a significant increase in Arabidopsis C_29_ Ket under NaCl stress. Both primary and secondary alcohols were identified in *B. carinata* leaf waxes. Primary alcohols accounted for 8% of the waxes accumulated on Arabidopsis leaves [34] and are the main wax class in camelina [25,26]. Primary alcohols showed an increase in soybean [29] and maritime pine [54] leaves under drought stress conditions.

Both cuticular wax biosynthesis pathways, acyl reduction (alcohol-forming pathway) and decarbonylation (alkane-forming pathway) [19,34,39,40], were identified in *B. carinata*. The abundance of alkane-forming pathway waxes (alkanes, ketones, and secondary alcohols) and their strong effects on total wax accumulation suggest that the decarbonylation pathway is the main pathway in *B. carinata*. Nonetheless, the acryl reduction pathway classes (primary fatty alcohols, wax esters, and free fatty acids) contributed to the variations in *B. carinata* leaf waxes. Tassone [42] obtained similar results in *B. napus* and suggested that both pathways are coupled in their effects on the production and accumulation of leaf waxes. The highly significant correlation coefficients between total wax and wax classes and components could indicate that these leaf wax compounds are dependently regulated, and selections for one wax class could affect another wax class. The C_29_ Alk, C_31_ Alk, C_29_ Alc-2, and C_29_ Ket are found to be the most significant wax components affecting the total leaf wax in *B. carinata*. These components had high heritability scores. These findings suggest that these components are heritable and strong potential targets for modifying *B. carinata* wax content and/or composition through selection during the early generations of *Brassica carinata* breeding programs for drought stress tolerance.

## 4. Materials and Methods

### 4.1. Plant Materials

A *Brassica carinata* diversity panel was assembled from 315 accessions collected from different parts of the world and stored at the USDA-ARS National Plant Germplasm System, Ames, IA, USA, the Leibniz Institute of Plant Genetics and Crop Plant Research, Gatersleben, Germany, and the Centre for Genetic Resources the Netherlands, Wageningen, The Netherlands (Supplementary Table S1). The accessions were planted in the greenhouse at the US Arid-Land Agricultural Research Center in Maricopa, Arizona at two different times: 22 March 2019, and 2 December, 2019. Accessions were arranged in a completely randomized design (CRD) with three replications each. The seeds of each accession were planted in two-gallon pots. Plants were regularly irrigated and fertilized. Leaf samples were collected from each pot at 35 days after planting. Each sample consisted of three middle leaves.

### 4.2. Wax Extraction and Analyses

Leaf waxes were extracted and analyzed following the protocols described in Tomasi et al. [25,26]. Waxes were extracted from each greenhouse and replicated separately. Immediately after harvesting leaf samples, the samples were submerged in 10 mL hexane (Sigma-Aldrich, St. Louis, MO, USA) and internal standards in a 20 mL glass scintillation vial. The wax extract vials were loaded onto the Agilent 7890A gas chromatograph, equipped with a 5975C mass spectrometer (Agilent, Santa Clara, CA, USA). Molecular identities of compounds were determined by the characteristic quadrupole electron impact mass spectra method. Leaf surface areas were calculated using ImageJ v1.53g software (https://imagej.net/ij/index.html, accessed 15 December 2020), and leaf waxes were calculated as µg dm^−2^.

### 4.3. Statistical Analyses

The Best Linear Unbiased Predictors (BLUPs) for each *B. carinata* accession for each wax component were estimated using SAS mixed linear models. The effects were partitioned into genetic effects, greenhouse effects (referred to as the environment), genotype x environment interactions (GxEs), and replication effects. The observed trait *Y* was analyzed as the response from the *i*th genotype in the *k*th replicate over the *j*th environment, using the model *Y_ijkl_ = μ + g_i_ + e_j_ + g_eij_ + r(e)_jk_ + error_ijkl_*, where *μ* is the grand mean, *g_i_* is the effect of the *i*th genotype, *e_j_* is the effect of the *j*th environment, *ge_ij_* is the interaction effect between the *i*th genotype and *j*th environment, and *r(e)_jk_* is the *k*th replicate effect nested in the *j*th environment.

Correlation Coefficients (rs) were calculated using PROC CORR of SAS software to assess the relationship among wax classes and compositions. Path analysis using Proc CALIS of SAS 9.4 software was used to partition the correlation coefficients for wax classes and compositions into direct and indirect effects contributing to total wax accumulation. The broad-sense heritability (H^2^) was calculated as: *H*^2^ = *σ*^2^*_G_*/(*σ*^2^*_G_ + σ*^2^*_error_/re + σ*^2^*_G×E_/e*), where *σ*^2^*_G_* is the genetic variance, *σ*^2^*_G×E_* is the genotype by environment interaction variance, *σ*^2^*_error_* is the residual error variance, *e* represents the number of environments, and *r* is the number of replicates [55,56,57].

## 5. Conclusions

The accumulation of leaf cuticular wax in plants can be one of the strategies to reduce non-stomatal water loss and thus, enhance resistance to abiotic stresses. A wide range of phenotypic variation in leaf total wax content and composition were characterized in the *B. carinata* diversity panel collected from different growing regions. The discovery of wide variations in leaf waxes is the first step in exploring wax biosynthetic pathways in *B. carinata*, moving toward identifying candidate genes that control wax accumulation, identifying its genetic networks, and developing molecular markers for molecular breeding and genomic selection programs to increase drought stress resistance in *B. carinata*. Among the detected waxes, C_29_ Alk (alkanes), C_29_ Alc-2 (secondary alcohol), and C_29_ Ket (ketone), products of the alkane forming pathway, were found to be the dominant waxes in all studied *B. carinata* accessions. These waxes had a high heritable nature. The possibility of identifying these waxes suggests their potential as good biomarkers in breeding for drought resistance in *B. carinata,* through modification of cuticle composition. The high heritability values of these waxes suggest the possibility of identifying candidate genes that control wax accumulation and selecting for these wax traits during the early generations of genetic improvement programs.

## Figures and Tables

**Table 1:** Means, ranges (min-max), and standard deviations (SDs) of BLUP values for total wax, wax classes, and components extracted from 315 *Brassica carinata* accessions.

Wax Components	Abr.	Min.	Max.	Mean	SD
Hexacosanoic acid (C_26_)	C_26_ FA	2.35	4.49	3	0.37
Octacosanoic acid (C_28_)	C_28_ FA	5.97	12.49	7.92	1.19
**Free Fatty Acids**		**8.44**	**16.7**	**10.81**	**1.45**
Heptacosane (C_27_)	C_27_ Alk	2.62	13.5	5.61	1.45
Octacosane (C_28_)	C_28_ Alk	2.88	8	3.7	0.45
Nonacosane (C_29_)	C_29_ Alk	90.48	518.82	283.21	95.55
Triacontane (C_30_)	C_30_ Alk	6.42	11.7	8.87	1.01
Hentriacotane (C_31_)	C_31_ Alk	37.85	226.34	96.17	35.4
Tritriacontane (C_33_)	C_33_ Alk	2.57	5.29	3.28	0.41
**Alkanes**		**144.88**	**636.5**	**403.8**	**114.84**
Tetracosanol (C_24_)	C_24_ Alc	4.55	5.9	5.06	0.25
Hexacosanol (C_26_)	C_26_ Alc	27.59	46.67	34.38	2.75
Octacosanol (C_28_)	C_28_ Alc	11.85	25.45	17.7	2.66
Nonacosanol (C_29_)	C_29_ Alc	1.33	3.95	2.26	0.51
Triacontanol (C_30_)	C_30_ Alc	3.81	10.83	5.35	0.93
**Primary Alcohols**		**47.97**	**85.69**	**63.74**	**6.63**
Nonacosan-(14)15-ol	C_29_ Alc-2	15.13	77.41	42.13	12.73
Triacontan-14(15)-ol	C_30_ Alc-2	3.92	9.28	6	1.11
Hentriacontan-14(15,16)-ol	C_31_ Alc-2	1.71	3.55	2.07	0.27
**Secondary Alcohols**		**16.47**	**86.74**	**50.1**	**15.55**
24-methyl-hexacosanol	mC_25_ Alc	0.97	3.18	1.65	0.42
24-methyl-hptacosanol	mC_26_ Alc	11.82	37.32	21.22	5.16
**Methyl Alcohols**		**12.5**	**40.41**	**22.85**	**5.69**
(C_43_) **Wax Ester**	C_43_ WE	7.28	7.94	7.44	0.09
Triacontanal (C_30_) **Aldehydes**	C_30_ Ald	19.91	51.67	27.42	4.92
Nonacosane-(14),15-one **Ketones**	C_29_ Ket	78.99	453.15	220.84	70.46
**Total Wax**		**289.31**	**1356**	**808.48**	**243.6**

## Data Availability

All data generated or analyzed during this study are available upon request to the corresponding author (Hussein Abdel-Haleem) at hussein.abdel-haleem@usda.gov.

## Supplementary Materials

The following supporting information can be downloaded at: https://www.mdpi.com/article/10.3390/plants12213716/s1; Table S1: List of the 315 Brassica carina accessions; Table S2: Correlation coefficients (r) of total wax, wax classes and components in *Brassica carinata* accessions, r values are above the horizontal axis, corresponding *p*-values are below the horizontal axis; Table S3: Direct (green) and indirect (black) effects of the total wax content related wax classes and components. Effect values are above the horizontal axis; corresponding *p*-values are below the horizontal axis.
